# Assessing the Current Public Knowledge, Attitudes, and Perceptions Regarding Consanguineous Marriages in the United Arab Emirates

**DOI:** 10.7759/cureus.106454

**Published:** 2026-04-05

**Authors:** Shahd N Alali, Jaafar H Hasan, Batool Musleh, Snds A Salama, Hind Alzaabi, Ahmad Alkhatib, Amal Hussein, Iman M Talaat

**Affiliations:** 1 Clinical Sciences Department, College of Medicine, University of Sharjah, Sharjah, ARE; 2 Family and Community Medicine, University of Sharjah, Sharjah, ARE; 3 Pathology, Faculty of Medicine, Alexandria University, Alexandria, EGY

**Keywords:** attitudes, consanguinity, cultural practices, hereditary diseases, knowledge, marriages, perceptions, premarital counseling, united arab emirates (uae)

## Abstract

Background and objective

Consanguinity refers to the marital practice between individuals who share blood relations. It is commonly observed in the Middle East, particularly through first-cousin unions. Previous studies have associated consanguineous marriages with an increased risk of genetic disorders and higher infant mortality rates. Much of the research conducted in the United Arab Emirates (UAE) on consanguinity is either outdated or does not specifically address the knowledge, attitudes, and perceptions of our target population. This study aimed to assess the knowledge, attitudes, and perceptions of UAE adults regarding consanguinity and its genetic consequences.

Methods

This was a cross-sectional study using convenience sampling to target UAE adult residents. A 37-question online survey was created, piloted, and then shared on social media platforms in both English and Arabic. The participants' knowledge, attitudes, and perceptions regarding consanguinity, premarital testing, and genetic disorders were assessed. Data were analyzed using SPSS Statistics version 26 (IBM Corp., Armonk, NY).

Results

The sample included 447 participants: 172 (38.5%) were men and 275 (61.5%) were women. Regarding nationality, 335 (74.9%) were non-local Arabs, 78 (17.4%) were Emirati nationals, and 34 (7.6%) were non-Arabs. Results show that 186 (41.6%) of participants had good knowledge, while 168 (37.6%) and 93 (20.8%) had moderate and poor knowledge, respectively. Higher knowledge scores were more common among participants with certain demographics, including women, non-local Arabs, healthcare sector employees, and individuals with non-related parents (p < 0.05). Regarding attitudes, 268 (60.0%) of participants had negative attitudes toward consanguinity. Negative attitudes were more frequent among participants with specific demographic characteristics, including female gender, younger age, non-local Arab ethnicity, students, unmarried individuals, and having non-related parents (p < 0.05). Additionally, 402 (90.0%) of participants believed that premarital testing is necessary.

Conclusions

This study shows that greater knowledge of the health consequences of consanguinity is associated with more negative attitudes toward the practice, indicating that awareness can influence societal perspectives. Demographic factors such as age, gender, and culture have a strong impact on attitudes, emphasizing the need for targeted interventions to shift perceptions of consanguineous marriages across different cultural groups.

## Introduction

A consanguineous marriage is a union between two people who are biologically related, particularly if they are second cousins or closer [1]. Approximately one billion people, or 20% of the world’s population, primarily in Muslim-majority countries in the Middle East, Africa, and South Asia, live in societies that favor consanguineous marriages. Pakistan has the highest rate, with 65% of marriages occurring between cousins, followed by Saudi Arabia (50%), Afghanistan (40%), Iran (30%), Egypt (20%), and Turkey (20%) [2]. This global practice has been traditionally maintained across many cultures for reasons such as preserving cultural values, maintaining family wealth, ensuring the geographical proximity of family members, honoring tradition, and strengthening familial bonds [3].

Consanguineous marriages have significant health impacts. Children of related parents are more likely to inherit autosomal recessive disorders because related parents are more prone to carry and pass on autosomal recessive genes to their offspring [4]. One of the most concerning health issues in the United Arab Emirates (UAE) is the prevalence of genetic abnormalities, primarily linked to consanguineous marriages. According to a 2012 news report, 75 out of 1000 children born in the UAE have congenital genetic disorders, with consanguineous marriages being a major risk factor [5]. Additionally, a study in Qatar found that children born to consanguineous parents are at higher risk of developing mental disorders, epilepsy, asthma, leukemia, and diabetes mellitus [4].

In the United Arab Emirates, earlier population-based studies reported that approximately 50% of marriages were consanguineous in 1997 [6]. More recently, a 2023 study among UAE nationals estimated a prevalence of 65% in an urban convenience sample [7]. These trends highlight the ongoing public health significance of consanguinity and its potential role in recessive genetic disorders, although the 2023 estimate may not fully represent the entire UAE population. Consanguineous marriages remain common throughout much of the Eastern world. A recent survey in the Kingdom of Saudi Arabia reported that 56% of all marriages, in both rural and urban areas, were consanguineous. Similar to Pakistan and Turkey, where links between low education levels and consanguineous marriages have been documented, high rates of consanguinity were also observed [8].

Understanding the public’s knowledge, attitudes, and perceptions regarding consanguineous marriages is crucial, as it helps address gaps in current research by providing up-to-date, locally relevant data that reflect contemporary trends and implications. While earlier studies mainly examined the prevalence and health effects of consanguineous marriages, little is known about how the general public perceives these practices today, as this area has been largely overlooked in previous UAE research due to cultural norms and the sensitivity of openly discussing family practices. Public health strategies and interventions are needed to mitigate the potential health risks of consanguineous marriages, aligning with recent national initiatives such as the Emirati Genome Programme, which profiles and sequences the genes of UAE citizens to enhance community health [5].

Premarital screening programs can significantly help reduce the risk of genetic diseases. However, even though these programs are mandatory, their effectiveness is often limited because couples identified as carriers of a recessive genetic disorder are still legally allowed to marry. Additionally, cultural and religious beliefs influence this practice, as some people believe their fate, including their children’s health, is predetermined by destiny, making them more willing to accept the risks associated with consanguineous marriages. Studying public attitudes toward consanguineous marriages can inform the development of health policies and educational campaigns aimed at reducing hereditary disorders. Research shows that attitudes significantly shape patterns of consanguineous marriages. For example, in Oman, the high rate of consanguineous marriages is viewed positively because it is associated with early marriage, early childbearing, and marital stability [9]. Similarly, in Syria, where data on consanguineous marriages were collected, the prevalence of these unions was influenced by favorable societal attitudes, especially among rural populations [10].

Consanguineous marriages remain common in many regions, including the UAE, and are associated with an increased risk of genetic disorders and congenital anomalies. Understanding public knowledge, attitudes, and perceptions regarding this practice is essential for developing effective public health strategies. The primary objective of this study was to assess the knowledge, attitudes, and perceptions of UAE adults regarding consanguinity and its genetic effects. Secondary objectives included evaluating awareness of genetic disorders in the UAE, examining knowledge of risk factors for congenital anomalies, assessing the willingness of UAE residents to undergo premarital genetic testing, analyzing the relationship between knowledge, attitudes, and perceptions, and exploring the influence of sociodemographic factors on these outcomes. Sociodemographic factors considered included age, gender, area of residence, ethnicity, occupation, education level, marital status, parental marital status, and the presence of consanguineous relationships within the family.

When discussing specific aspects of knowledge, attitudes, and perceptions within the community, this study examines factors influencing knowledge about consanguineous marriages, including awareness of associated genetic risks. It evaluates whether community members understand the link between consanguinity and genetic disorders, including congenital anomalies, as well as their relationship with infant mortality rates. Participant knowledge levels were categorized as poor, moderate, or good based on responses to questions about the health risks associated with consanguinity, helping to identify areas where targeted awareness campaigns may be most needed.

The study also examines participants’ attitudes toward consanguineous marriages, identifying factors that shape these views, such as personal experiences, cultural influences, and educational background. Perceptions are further assessed by evaluating how participants view the health risks associated with consanguinity and how their education, cultural context, and access to information may influence these perceptions.

A review of the literature shows that the latest studies on consanguinity in the UAE are either outdated or not specific to the knowledge, attitudes, and perceptions of UAE adult residents toward consanguinity. Most other studies focused primarily on the prevalence of consanguineous marriages across Arab countries, including the UAE, without examining public perceptions [11]. Similarly, another UAE study highlighted the health outcomes of consanguineous unions but did not explore awareness or attitudes among the general population [12]. Comparable studies have been conducted in neighboring countries like Saudi Arabia, which have similar rates of consanguinity, but there has been no recent research on this topic in the UAE. This reveals a clear gap in the literature, which our study aims to address with a recent, comprehensive investigation.

The importance of this study lies in its potential impact on public health, policy, and education. The findings can help shape policies that address the cultural and educational needs of communities and provide a foundation for future research in this area. Additionally, our study has several unique objectives compared to previous research in the UAE. Unlike many studies that focus mainly on the prevalence and genetic consequences of consanguinity, our research emphasizes the public’s attitudes toward consanguineous practices, including premarital testing. This focus on premarital testing as a preventive measure represents a distinct perspective not commonly addressed in other studies. Another unique feature of this study is its exploration of how cultural background and personal experiences, such as having consanguineous parents, influence participants’ views on these marriages. This aspect is rarely addressed in prior research, which often examines the general population without considering individual family histories.

## Materials and methods

Study design, setting, and population

This study employed a descriptive, cross-sectional design targeting the adult resident population of the UAE. The aim was to evaluate participants’ knowledge of consanguinity and its potential health effects, as well as their attitudes and perceptions toward this common cultural practice. Data were collected using a bilingual (Arabic and English), piloted, self-administered online questionnaire. The inclusion criteria were all adult residents of the UAE who had access to social media, could read and understand English or Arabic, and were willing to participate. Individuals living outside the UAE, tourists in the UAE, and those under 18 years of age were excluded.

The survey link was distributed through various social media platforms, including Facebook, Instagram, Telegram, and WhatsApp, using a non-probability convenience sampling method. Groups with diverse interests were included, such as the general community, family, and educational groups. Participants were encouraged to share the survey with other adult UAE residents. No demographic targeting was applied beyond residency eligibility. To prevent duplicate responses, participants were asked to provide the first three letters of their family name and the last three digits of their phone number, and any duplicates were removed during data cleaning. A response rate could not be calculated because the survey was distributed across multiple social media platforms and group channels, making the total number of individuals reached indeterminable. Data collection was conducted between March and April 2023.

Data collection tool

The questionnaire used for data collection was developed based on a review of relevant literature examining knowledge, attitudes, and perceptions toward consanguineous marriages. Due to the limited availability of validated survey instruments on this topic, the authors designed a structured questionnaire specifically tailored to the objectives of this study. Previously published studies conducted in other Gulf countries, such as Qatar [13] and Saudi Arabia [14], served as the primary references because of their cultural and social similarities to the UAE. Additional concepts and question formats were drawn from studies conducted in South Asian countries [15,16]. Some items were developed specifically by the authors to address the study objectives, while others were adapted and modified to ensure clarity and cultural relevance for UAE residents.

The final questionnaire (see Appendix A) consisted of 37 items divided into four sections: initial inclusion criteria (two questions) and demographics (nine questions), knowledge (13 questions), attitudes (seven questions), and perceptions (six questions). The survey was initially developed in English and subsequently translated into Arabic. Translation was performed independently by two authors, and their versions were compared and reconciled to produce a single final version. The Arabic version was reviewed and approved by the research supervisors and the Research Ethics Committee of the University of Sharjah to ensure clarity, cultural relevance, and content validity.

The demographics section included participants’ age, gender, ethnicity, educational background, and occupation. Additional questions asked about marital status, current or previous involvement in a consanguineous marriage, and the nature of their parents’ relationship. The knowledge section included closed-ended questions with three response options: yes, no, and I don’t know. The first two questions assessed participants’ understanding of the terms ‘consanguineous marriage’ and ‘premarital testing.’ A definition was provided afterward to ensure participants had sufficient knowledge to understand the remaining questionnaire. The next 11 questions assessed participants’ awareness of the health consequences of consanguinity, risk factors for genetic conditions, and related topics.

The attitudes section consisted of seven questions, each rated on a 5-point Likert scale (strongly agree, agree, neutral, disagree, strongly disagree). It presented various scenarios related to consanguinity to evaluate participants’ behaviors and opinions regarding the practice. In scoring, items were reversed when higher agreement indicated a negative attitude, so that higher total scores consistently reflected more positive attitudes. The perceptions section included five questions, also using a 5-point Likert scale, to assess participants’ opinions and preconceptions about consanguinity. This was followed by a multiple-select question, allowing respondents to choose more than one option regarding the perceived benefits of consanguinity, if any.

The questionnaire was piloted on 14 individuals to verify the accuracy of the Arabic translation, clarity of questions in both languages, and time required to complete the survey. A paragraph containing the Participant Information Sheet (PIS), explaining the study’s purpose, eligibility criteria, and contact details for communication with the researchers, was included with the survey link to ensure transparency. Participants were informed that the study was conducted by second-year medical students at the University of Sharjah to explore knowledge, attitudes, and perceptions regarding consanguineous marriages among adults in the UAE.

Participation was entirely voluntary, and respondents were aware that they could decline participation or withdraw at any time before submitting the questionnaire. The survey took approximately five to seven minutes to complete and posed no risk to participants. All responses were collected anonymously, and withdrawal was not possible after submission. Collected information was kept strictly confidential and used solely for research purposes. The questionnaire was administered via Google Forms, which included mandatory questions to ensure completeness and logic skips to present questions only when relevant (e.g., unmarried participants skipped items about marital history and consanguineous marriage involvement).

Sample size calculation

Because there is limited data on knowledge, attitudes, and perceptions regarding consanguineous marriages in the UAE, sample size calculations were based on recent studies conducted in the Gulf region, specifically Saudi Arabia [17,18]. For statistical values not reported in the literature, the prevalence was assumed to be 50%. The formula used to estimate a single proportion was the Cochran's formula: $n = \frac{Z^2 \ p (1-p) }{e^2}$, where n indicates the minimum number of participants required for the study, p corresponds to the expected prevalence of the outcome of interest, Z represents the standard normal deviate associated with the chosen confidence level (1.96 for 95% confidence), and e reflects the permissible margin of error for the estimate. After accounting for missing data, the highest calculated sample size was used, resulting in a target of 424 participants with a 5% alpha level. This provides a 95% confidence interval (CI). Given the exploratory and descriptive nature of this study, this sample size provides sufficient statistical power to detect meaningful differences in knowledge, attitudes, and perceptions, and no additional formal power calculation was required.

Statistical analysis

Statistical analysis was conducted using IBM SPSS for Windows, Version 26 (Released 2018; IBM Corp., Armonk, NY). A total of 467 responses were received, exceeding the minimum target. Missing data were managed by including only complete responses for each domain in the analysis. For variables with partially missing responses, valid percentages were reported to ensure that analyses reflected only the available data. This approach minimizes bias from incomplete responses without imputing values, which could otherwise artificially influence the results. After removing duplicates and handling missing data, 447 eligible responses were included in the final analysis.

Knowledge Scoring

Responses were assigned numerical values, with most questions worth one point and four essential questions worth two points (marked with a star in Appendix A). Total knowledge scores were calculated by summing all item responses (maximum score = 17). Participants were categorized as having poor knowledge (0-10 points), moderate knowledge (11-15 points), or good knowledge (16-17 points). These thresholds were determined through discussion among the authors and supervisors, considering the weighting of essential questions and the total possible score, to reflect meaningful distinctions in participant knowledge levels.

Attitude Scoring

Each of the seven attitude items was rated on a five-point Likert scale. Scores were reversed for items where agreement indicated a negative attitude, ensuring that higher scores consistently reflected more positive attitudes. Total scores were summed, and participants were categorized as having a positive attitude (7-20 points) or a negative attitude (21-35 points). Cutoffs were established by the authors to distinguish participants expressing overall agreement versus disagreement with consanguineous marriages. For analysis purposes, neutral responses on the Likert scale were included with positive attitudes, as they do not indicate disagreement with the practice.

All study variables, including demographics, knowledge, attitudes, and perceptions, were initially analyzed using univariate descriptive statistics and summarized as frequencies and percentages. Bivariate analyses were then performed to assess associations between variables using the Chi-square (χ²) test. Odds ratios (ORs) with 95% CIs were calculated for selected bivariate associations to estimate the strength of the relationships between variables. All demographic variables were tested for associations with knowledge and attitude categories. Bivariate analyses were also performed to examine the relationship between knowledge levels and attitudes.

Additionally, among married participants, knowledge and attitude scores were compared between those in consanguineous versus non-consanguineous marriages using the χ² test. A p-value of ≤0.05 was considered statistically significant. Since all variables analyzed were categorical and the study was primarily descriptive, multivariable regression modeling was not performed to avoid unstable estimates. Furthermore, because multiple bivariate comparisons were performed in an exploratory context, no formal adjustment for multiple testing was applied, and results were interpreted cautiously. Internal consistency reliability was evaluated using Cronbach’s alpha, yielding acceptable values for the knowledge (α = 0.795) and attitude (α = 0.777) domains. The perception domain was analyzed descriptively only.

Ethical considerations

The researchers adhered to the highest ethical standards, following the principles outlined in the Declaration of Helsinki throughout the study. The Research Ethics Committee at the University of Sharjah thoroughly reviewed the research tools and objectives, and approval was obtained before the pilot study (reference number: REC-23-02-18-05-S). The Participant Information Sheet (PIS) attached to the questionnaire link clearly explained the purpose of the research and the inclusion criteria, ensuring informed consent before enrollment. Completion and submission of the questionnaire were considered implied consent, and all participants voluntarily participated. No identifying information was collected. Responses were kept anonymous, and the data were stored in a password-protected database accessible only to the research team. The published results do not reveal the identities of any study participants.

## Results

Demographics

As shown in Table 1, 447 valid responses were retained; 172 (38.5%) were males, and 275 (61.5%) were females. Over half 238 (53.2%) of the participants were between 18 and 29 years old. Most participants (335, 74.9%) were non-local Arabs, with 258 (57.7%) participants residing in the Northern Emirates. Additionally, 306 (68.5%) held a higher education qualification, such as a diploma or undergraduate degree. Less than half (195, 43.6%) were students, and 85 (19%) were unemployed. Among the currently married participants (198, 44.3%), 58 (29.3%) were married to a blood-related relative, while the remaining were not. About a third of the participants' parents were blood relatives (162, 36.3%).

**Table 1:** Demographic and social factors (n = 447)

Demographic factors	Options	Frequency	Percentage (%)
Gender	Male	172	38.5
	Female	275	61.5
Age group, years	18-29	238	53.2
	30-49	159	35.6
	50+	50	11.2
Nationality	Local	78	17.4
	Non-local Arab	335	74.9
	Non-Arab	34	7.6
Emirate of residence	Northern Emirates	258	57.7
	Dubai	100	22.4
	Abu Dhabi	89	19.9
Level of education	High school or less	92	20.6
	Diploma and undergraduate level	306	68.5
	Post-graduate	49	11.0
Occupational sector	Student	195	43.6
	Healthcare	41	9.2
	Other sector	126	28.2
	Unemployed	85	19.0
Marital status	Unmarried	249	55.7
	Married	198	44.3
Relationship with current/previous spouse	First cousins	26	5.8
	Other relative	34	7.6
	Not related	151	33.6
	Not applicable (single)	236	53.0
Parents' relationship	First cousins	75	16.8
	Second cousins	87	19.5
	Not related	285	63.8

Knowledge about consanguinity

Table 2 presents the questions and responses in the knowledge section of the survey. About half of the participants (202, 45.2%) were unaware that consanguineous marriages can increase the risk of auditory and visual deficiencies in newborns. Infectious diseases were the least recognized risk factor for birth defects, with 140 participants (31.3%) unaware of their association. In contrast, consanguinity itself was identified as a risk factor by 363 participants (81.2%), making it the third most commonly recognized risk factor, following medication use during pregnancy, reported by 401 participants (89.7%), and tobacco and alcohol use, reported by 396 participants (88.6%). Additionally, 344 participants (77%) recognized that the degree of kinship is positively associated with an increased likelihood of passing on hereditary diseases. More than half of the participants (233, 52.1%) were unaware that consanguineous marriages are linked to higher abortion rates. When knowledge scores were categorized, 186 participants (41.6%) demonstrated good knowledge, 168 participants (37.6%) had moderate knowledge, and 93 participants (20.8%) were classified as having poor knowledge.

**Table 2:** Knowledge questions related to genetic disorders and health risks of consanguinity (n = 447)

Knowledge items		Correct		Incorrect	
		N	%	N	%
Heard of:	Consanguinity	370	82.8	77	17.2
	Premarital testing	412	92.2	35	7.8
Increased risk with consanguinity:	Thalassemia	318	71.1	129	28.8
	Hearing and visual abnormalities	245	54.8	202	45.2
	Down syndrome	282	63.1	165	36.9
	Intellectual disability	293	65.5	154	34.5
Increases the risk of inherited diseases:	Consanguinity	363	81.2	85	18.4
	Medications	401	89.7	46	10.3
	Tobacco and alcohol use during pregnancy	396	88.6	51	11.4
	Infectious diseases	307	68.7	140	31.3
Do you think that the risk of passing down inherited diseases is less in marriages between distant relatives when compared to marriages between close relatives?		344	77	103	23
Do you think that consanguinity causes congenital anomalies?		362	81	85	19
Do you think that consanguineous marriages are associated with a higher abortion rate?		214	47.9	233	52.1

Attitudes toward consanguinity

**Figure 1:**
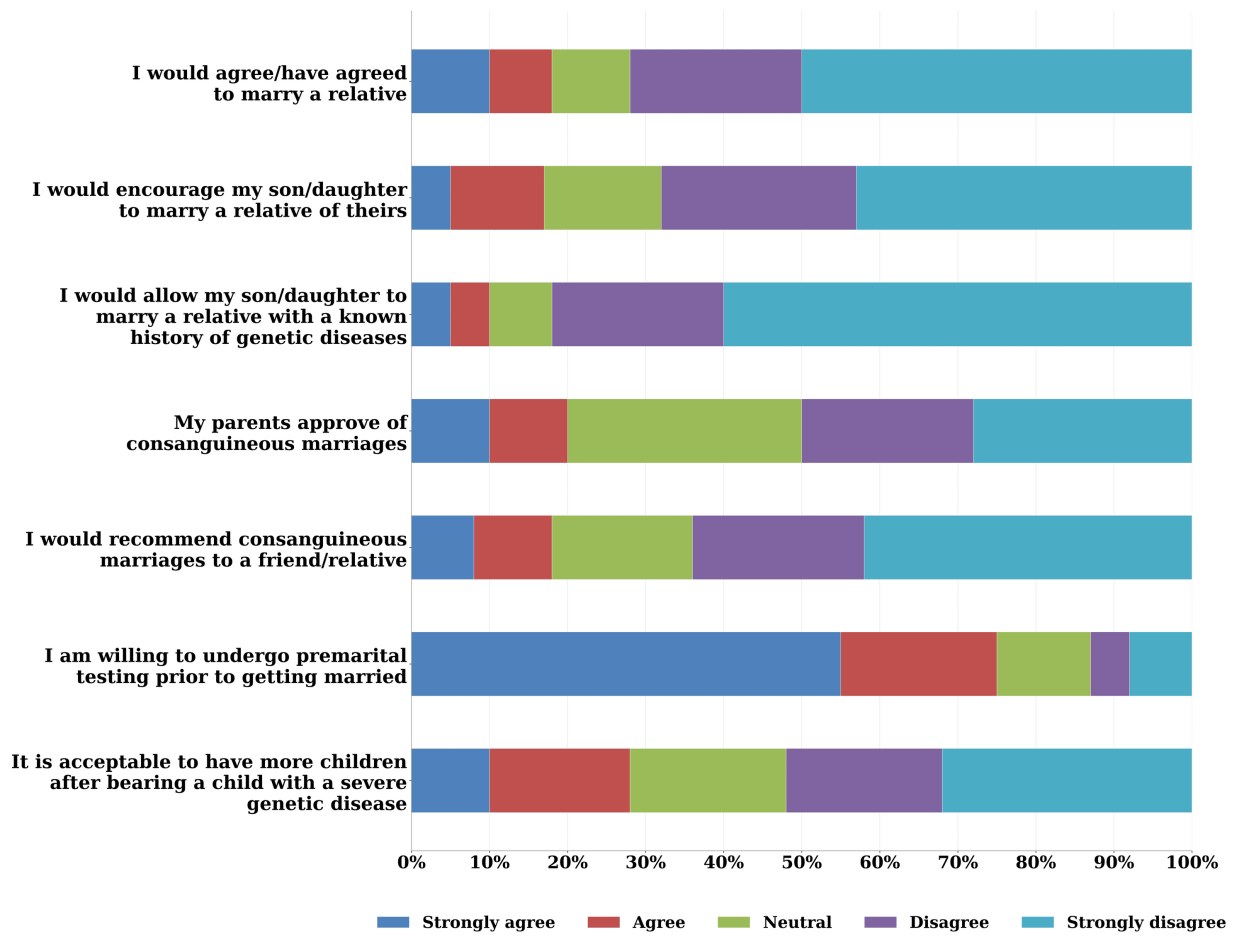
Participants’ attitudes toward consanguinity measured on a 5-point Likert scale The figure presents a stacked horizontal bar chart showing participants’ responses to seven questions assessing attitudes towards consanguineous marriages, measured on a 5-point Likert scale ranging from "strongly agree" to "strongly disagree"

As shown in Figure 1, attitudes were evaluated using a 5-point Likert scale ranging from "strongly agree" to "strongly disagree". The majority of the sample (358, 80%) indicated a willingness to undergo premarital testing. While participants’ parents were generally more supportive of consanguineous marriages, most participants themselves opposed recommending such unions (270, 60.5%) or encouraging their children to enter into them (260, 58.2%). Most of the sample (268, 60.0%) held negative attitudes toward consanguinity (i.e., did not support it), while the remaining (179, 40.0%) held positive views.

Perceptions regarding consanguinity

**Figure 2:**
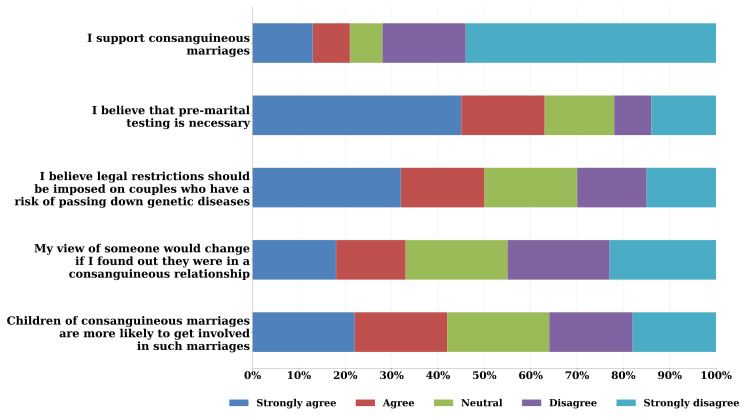
Participants’ perception regarding consanguinity measured on a 5-point Likert scale The figure presents a stacked horizontal bar chart showing participants’ responses to five questions assessing perceptions of consanguineous marriages, measured on a 5-point Likert scale ranging from "strongly agree" to "strongly disagree"

Regarding perceptions (Figure 2), nearly half of the sample (224, 50.1%) disagreed with the statement “I support consanguineous marriages,” and 259 (58.0%) agreed or strongly agreed with implementing legal restrictions on couples at risk of passing down genetic diseases. The vast majority (406, 90.9%) believed that premarital testing was necessary, and 187 (41.9%) thought that children of consanguineous marriages were more likely to enter into such marriages themselves.

Table 3 presents the results of a multiple-choice question regarding the perceived benefits of consanguineous marriages. The most commonly chosen benefit was that the "bride or groom knew the family circumstances", with 252 (56.5%) of the sample selecting it. This was followed by "promoting tradition and continuity", 167 (37.4%). About a third of the sample, 125 (28.0%), believed that consanguineous marriages have no benefits.

**Table 3:** Advantages of consanguineous marriages (n = 447) Participants were allowed to select multiple options; hence, the percentages do not necessarily add up to 100%

Advantage	N	%
The bride and groom are familiar with the family circumstances	252	56.40%
The financial status is more stable	134	30%
Promotes tradition and continuity	167	37.40%
The marriage is more harmonious and loving	84	18.80%
It is easier to raise the children	83	18.60%
If there are any disorders, they are known and expected	115	25.70%
Consanguineous marriages have no benefits	125	28%

Association between knowledge and attitude levels

Table 4 displays the relationships between knowledge and attitude levels and sociodemographic factors. Bivariate analysis results showed that the factors significantly associated with higher knowledge levels were female gender (χ² = 10.285, p = 0.006), non-local Arab nationality (χ² = 21.654, p < 0.001), occupation in the healthcare sector (χ² = 21.201, p = 0.02), being married to a non-relative (χ² = 26.912, p < 0.001), and having parents who are not related (χ² = 20.593, p < 0.001). Negative attitudes were significantly associated with female gender (χ² = 4.204, p = 0.04), younger age groups (χ² = 16.004, p < 0.001), non-local Arab nationality (χ² = 7.639, p = 0.022), high school or lower education levels (χ² = 8.247, p = 0.016), being a student (χ² = 15.043, p = 0.02), being unmarried (χ² = 13.335, p < 0.001), and parents not being related (χ² = 26.006, p < 0.001). Additionally, higher knowledge levels were significantly linked to negative attitudes toward consanguinity (χ² = 25.382, p < 0.001). This association was further supported by odds ratio analysis, which indicated that participants with good knowledge were almost twice as likely to exhibit negative attitudes compared with those with poor or moderate knowledge (OR: 1.97, 95% CI: 1.33-2.92).

**Table 4:** Bivariate analysis between participants’ demographic and social characteristics and their knowledge and attitude levels regarding consanguinity (n = 447) Associations were assessed using the Pearson chi-square test (χ²), and corresponding p-values are reported

Variables	Poor knowledge		Moderate knowledge		Good knowledge		χ²	P-value	Positive attitudes		Negative attitudes		χ²	P-value
	N	%	N	%	N	%			N	%	N	%		
Gender														
Male	49	28.5	56	32.6	67	39.0	10.285	0.006	80	46.5	92	53.5	4.204	0.040
Female	44	16.0	112	40.7	119	43.3			101	36.7	174	63.3		
Age group, years														
18-29	48	20.2	86	36.1	104	43.7	1.453	0.835	79	33.2	159	66.8	16.004	<0.001
30-49	36	22.6	61	38.4	62	39.0			71	44.7	88	55.3		
50+	9	18.0	21	42.0	20	40.0			31	62.0	19	38.0		
Nationality														
Local	22	28.2	29	37.2	27	34.6	21.654	<0.001	42	53.8	36	46.2	7.639	0.022
Non-local Arab	55	16.4	129	38.5	151	45.1			124	37.0	211	63.0		
Non-Arab	16	47.1	10	29.4	8	23.5			15	44.1	19	55.9		
Emirate of residence														
Northern Emirates	62	24.0	100	38.8	96	37.2	8.156	0.086	105	40.7	153	59.3	0.293	0.864
Dubai	17	17.0	40	40.0	43	43.0			42	42.0	58	58.0		
Abu Dhabi	14	15.7	28	31.5	47	52.8			34	38.2	55	61.8		
Level of education														
High school or less	12	13.0	34	27.0	46	50.0	7.027	0.134	33	35.9	59	64.1	8.247	0.016
Diploma and undergraduate	67	21.9	119	38.9	120	39.2			119	38.9	187	61.1		
Post-graduate	14	28.6	15	30.6	20	40.8			29	59.2	20	40.8		
Occupational sector														
Student	36	18.5	71	36.4	88	45.1	21.201	0.002	59	30.3	136	69.7	15.043	0.002
Healthcare	4	9.8	10	24.4	27	65.9			20	48.8	21	51.2		
Other sector	37	29.4	52	41.3	37	29.4			61	48.4	65	51.6		
Unemployed	16	18.8	35	41.2	34	40.0			41	48.2	44	51.8		
Marital status														
Unmarried	49	19.7	90	36.1	110	44.2	1.542	0.462	82	32.9	167	67.1	13.335	<0.001
Married	44	22.2	78	39.4	76	38.4			99	50.0	99	50.0		
Relationship with current/previous spouse														
First cousins	15	57.7	6	23.1	5	19.2	26.912	<0.001	19	73.1	7	26.9	32.593	<0.001
Other relative	9	26.5	11	32.4	14	41.2			25	73.5	9	26.5		
Not related	22	14.6	65	43.0	64	42.4			59	39.1	92	60.9		
Not applicable (single)	47	19.9	86	36.4	103	43.6			78	33.1	158	66.9		
Parents' relationship														
First cousins	28	37.3	18	24.0	29	38.7	20.593	<0.001	43	57.3	32	42.7	26.006	<0.001
Other relative	20	23.0	38	43.7	29	33.3			48	55.2	39	44.8		
Not related	45	15.8	112	39.3	128	44.9			90	31.6	195	68.4		

Determinants of consanguineous marriage in the study sample

Table 5 illustrates the associations between the prevalence of consanguineous marriages in our sample and various factors. Among married participants, consanguineous marriages were significantly more common among UAE citizens (χ² = 14.007, p = 0.001) and participants whose parents were first cousins (χ² = 23.145, p < 0.001). Other factors associated with consanguineous marriages included possessing a poor level of knowledge (χ² = 18.422, p < 0.001) and having positive attitudes toward consanguinity (χ² = 16.895, p < 0.001). Regarding the strength of these associations, participants whose parents were first cousins had an odds ratio of 4.06 (95% CI: 1.88-8.73), indicating a higher likelihood of entering a consanguineous marriage compared with participants whose parents were not first cousins. Similarly, being a UAE citizen was associated with an odds ratio of 5.25 (95% CI: 2.06-13.34), reflecting the influence of cultural practices on participation in consanguineous marriages.

**Table 5:** Bivariate analysis between type of marriage and participants’ demographic characteristics, knowledge levels, and attitude levels regarding consanguinity (n = 198) Associations were evaluated using the Pearson chi-square test (χ²), and corresponding p-values are reported

Variables	Consanguineous marriage		Non-consanguineous marriage		χ²	P-value
	N	%	N	%		
Gender						
Male	22	25.3	65	74.7	1.095	0.295
Female	36	32.4	75	67.6		
Age group, years						
18-29	5	38.5	8	61.5	0.600	0.741
30-49	40	28.2	102	71.8		
50+	13	30.2	30	69.8		
Nationality						
Local	14	63.6	8	36.4	14.007	0.001
Non-local Arab	39	24.5	119	75.5		
Non-Arab	5	27.8	13	72.2		
Emirate of residence						
Northern emirates	31	26.1	88	73.9	2.500	0.286
Dubai	14	29.8	33	70.2		
Abu Dhabi	13	40.6	19	59.4		
Level of education						
High school or less	5	31.3	11	68.8	0.794	0.672
Diploma and undergraduate	40	27.6	105	72.4		
Post-graduate	13	35.1	24	64.9		
Parents relationship						
First cousins	19	55.9	15	44.1	23.145	<0.001
Other relative	19	41.3	27	58.7		
Not related	20	16.9	98	83.1		
Knowledge level						
Poor	24	55.8	19	44.2	18.422	<0.001
Moderate	17	21.8	61	78.2		
Good	17	22.1	60	77.9		
Attitude						
Positive	42	42.9	56	57.1	16.895	<0.001
Negative	16	16.0	84	84.0		

## Discussion

This cross-sectional study examined knowledge, attitudes, and perceptions regarding consanguineous marriages among the UAE population. The findings reveal a gap between awareness of the genetic risks associated with consanguinity and the prevalence of positive attitudes toward these unions. Despite high recognition of the importance of premarital genetic testing and understanding of specific health risks, a notable proportion of participants expressed favorable opinions toward consanguineous marriages. These observations underscore the potential benefit of targeted educational programs to provide information on genetic risks and promote informed decision-making among young adults in culturally sensitive contexts.

Knowledge about consanguinity

In this study, 41.6% of respondents demonstrated good knowledge about consanguinity, compared to 9% in a similar study from Saudi Arabia [8]. Most respondents (81%) recognized that consanguineous marriage can be associated with congenital anomalies, aligning with findings from another UAE study targeting students [19]. Lower awareness levels have been reported in studies from India [20] and Bangladesh [21], where only 3.5% and 7% of participants were aware, respectively. These observations suggest that knowledge in the UAE may be relatively higher, potentially reflecting the population’s educational level. Participants in our study were also more aware of the link between consanguineous marriage and thalassemia compared to the Saudi Arabia study [8]. The least recognized health effects in our sample were the increased risks of abortion and auditory or visual impairments in offspring.

Sociodemographic factors influencing knowledge

Observed associations in our sample indicate that gender, nationality, occupational sector, relationship with a spouse, and parents’ relationship were linked to differences in knowledge levels. Female gender, non-local Arab nationality, employment in the healthcare sector, being married to a non-relative, and having unrelated parents were all associated with higher levels of knowledge. Mahboub’s 2018 study in Saudi Arabia found similar patterns, with lower knowledge scores among males and those married to relatives [8]. In contrast, Jairoun et al. reported that individuals with a family history of consanguineous marriages tended to have higher knowledge scores [19], implying that personal or family experience may influence awareness of associated risks.

Prevalence of consanguinity and associated factors

In our sample, the prevalence of consanguinity differed according to several factors, including UAE citizenship, having first-cousin parents, lower knowledge levels, and reported positive attitudes toward consanguinity. Similarly, other studies have noted higher participation in consanguineous marriages among individuals with lower knowledge of genetic risks or with related parents [22]. These are observed associations and should not be interpreted as indicating causation.

Attitudes toward consanguinity

Most participants in our study (86.35%) favored premarital genetic testing. About 60.5% expressed negative attitudes toward consanguineous marriages, and 58.2% opposed their children marrying a relative. However, 33.6% reported parental support for consanguinity, indicating variation across generations, and 71.48% would discourage their children from marrying someone with a known hereditary condition. Higher knowledge levels were observed among participants with negative attitudes toward consanguinity (p ≤ 0.001), and higher education levels and younger age groups were also associated with negative attitudes (p ≤ 0.001). These findings are comparable to other UAE research [7], which found a significant association between awareness of genetic risks and negative attitudes (p ≤ 0.001). Support for genetic counseling in our sample (86.35%) was higher than that reported by Jairoun et al. (65%) [19]. Consistent with prior studies, females were more likely to report negative attitudes than males, and positive attitudes toward consanguinity were more frequent among students from rural backgrounds in a Saudi study (p = 0.03) [23].

Younger, unmarried individuals and students tended to oppose consanguineous marriages, likely due to cultural differences, education, and the fact that they had not experienced marriage themselves. Participants with consanguineous parents had more positive attitudes, possibly due to prior exposure. There is a correlation between higher knowledge and negative attitudes, presumably due to awareness of the harmful effects of consanguinity. Non-local Arabs showed greater knowledge, which may be influenced by information accessibility and cultural differences. Individuals with unrelated parents displayed more awareness, indicating the impact of personal experiences on knowledge levels. Healthcare professionals exhibited a deeper understanding of the health-related consequences of consanguinity, reflecting their professional expertise.

Implications for public health and genetic counseling

Concerns about genetic disorders were frequently cited as reasons for opposing consanguineous unions. In Saudi studies, 44.2% of participants opposed consanguineous marriages due to increased genetic risks [24], and 57.6% were aware of potential hereditary risks, with higher awareness observed alongside negative attitudes [8]. In our study, over three-quarters of participants perceived a lower risk in marriages between distant relatives compared with close relatives and reported reluctance to recommend such marriages for their offspring when hereditary conditions were known. These patterns suggest that awareness of genetic risks may be associated with more cautious attitudes. Cross-sectional data cannot determine whether knowledge causes these attitudes, but the observed patterns may inform the design of culturally sensitive educational programs that provide relevant information on potential health risks.

Limitations

This study has several limitations to consider. First, the use of non-probability convenience sampling through online platforms likely introduced sampling bias, as younger, female, and digitally active participants were overrepresented. Consequently, the results may not be generalizable to the broader UAE population, particularly older adults, rural residents, or those with limited internet access. Additionally, because the UAE is highly multicultural and multilingual, administering the survey only in Arabic and English may have further restricted participation, affecting representativeness. Since the survey was distributed through multiple social media channels, the total number of individuals reached could not be determined, and a response rate could not be calculated, limiting the assessment of potential non-response bias.

The questionnaire was newly developed and has not undergone full psychometric validation. While internal consistency was acceptable for the knowledge and attitude domains, formal assessments of face validity, content validity, test-retest reliability, and full psychometric evaluation were not performed, which should be addressed in future research. To aid replicability, essential knowledge questions are marked in Appendix A, and scoring rules are provided.

As an online self-reported survey, responses may be influenced by reporting or social desirability bias. Individuals less familiar with technology, particularly older adults, may have been underrepresented, as reflected in the small number of responses from older age groups. Their perspectives are important, as cultural practices such as consanguineous marriage can differ by age and tradition. Finally, the cross-sectional design prevents causal inferences, and multivariable analysis was not performed, so confounding factors could not be controlled. Therefore, findings should be viewed as exploratory and indicative rather than fully representative. Future studies with more representative sampling and validated instruments are warranted to guide public health interventions.

Strengths

Despite these limitations, the study has several methodological strengths. The descriptive cross-sectional design with a sample size of 447 exceeded the calculated minimum, providing sufficient power for exploratory analyses. The bilingual questionnaire (Arabic and English) was piloted and showed acceptable internal consistency (Cronbach’s α = 0.795 and 0.777 for knowledge and attitude, respectively), with essential knowledge items clearly marked in Appendix A to improve transparency and replicability. Appropriate descriptive and bivariate statistics were used to explore associations between demographics, knowledge, and attitudes.

This study is the most recent of its kind in the UAE, capturing knowledge, attitudes, and perceptions across a diverse, multicultural population. By addressing all three domains, it provides a comprehensive snapshot of public views on consanguinity, which can serve as a baseline for future research. The study’s specific objectives were achieved using a purpose-built questionnaire in the absence of a validated tool, offering exploratory insights that can inform future longitudinal studies, interventions, and culturally sensitive public health programs.

## Conclusions

Understanding consanguinity, its health consequences, and attitudes toward the practice is influenced by various factors, including age, cultural background, and educational level. Encouragingly, our findings show a reduction in positive perceptions of consanguineous relationships. However, the continued support for such unions among a substantial proportion of participants highlights the need for targeted, large-scale educational initiatives. The factors associated with knowledge can help guide awareness programs tailored to specific populations, particularly those in which the practice is more prevalent. These programs may emphasize the genetic and health risks associated with consanguinity, which are important for both patient well-being and public health. This cross-sectional study provides a current baseline of knowledge, attitudes, and perceptions in the UAE. Future research using more representative sampling strategies, fully validated questionnaires, and longitudinal or multivariable approaches is warranted to generate more robust evidence that can better inform public health initiatives and policy decisions.

## Appendices

Appendix A

**Table 6:** English version of the questionnaire Complete English version of the survey instrument used in the study, including items related to inclusion criteria, demographic characteristics, knowledge, attitudes, and perceptions regarding consanguinity. Questions marked with a * are essential knowledge questions, as described in the Methods section

Section	Item number and question Type	Questions/instructions		Choices
Inclusion and exclusion	1. Single choice	Are you currently residing in the UAE?		Yes
				No
	2. Single choice	Are you above the age of 18?		Yes
				No
Demographics	3. Single choice	Please select your gender:		Male
				Female
	4. Single choice	Please specify your age group:		18-29
				30-39
				40-49
				50-59
				60+
	5. Single choice	Please select what applies to you:		Local
				Non-local Arab
				Non-Arab
	6. Single choice	Select your Emirate of residence:		Sharjah
				Dubai
				Abu Dhabi
				Ajman
				Umm Al Quwain
				Ras Al Khaimah
				Fujairah
	7. Single choice	Please specify your highest level of education:		No education
				Elementary school level
				Middle school level
				Secondary/high school level
				Diploma level
				Undergraduate level (bachelor’s degree)
				Post-graduate (master’s or higher)
	8. Single choice	Please specify your occupational sector/status:		Student
				Healthcare
				Social/welfare work
				Mechanic/engineer
				Housekeeping/garbage and waste management
				Retail
				Construction
				Delivery services
				Education and research
				Unemployed
				Housewife
				Retired
				Other
	9. Single choice	Please specify your marital status:		Single
				Married
				Divorced
				Widowed
	10. Single choice	Specify your relationship with your current/previous spouse:		First cousins
				Second cousins
				Distant relatives (e.g., third cousin, etc.)
				Not related
				Not applicable (single)
	11. Single choice	Specify your parents’ relationship:		First cousins
				Second cousins
				Distant relatives (e.g., third cousin, etc.)
				Not related
Knowledge	12. Single choice	Have you heard of the term consanguinity?		Yes
				No
	13. Single choice	Have you heard of the term premarital testing?		Yes
				No
	14. Single choice	Do you think that consanguinity causes congenital anomalies?*		Yes
				No
				I don’t know
	15. Single choice	Do you think that consanguineous marriages are associated with a higher abortion rate?		Yes
				No
				I don’t know
	16. Single choice	Do you think the risk of the following inherited diseases increases with consanguinity?	Thalassemia*	Yes
				No
				I don’t know
			Intellectual disability	Yes
				No
				I don’t know
			Hearing and visual disabilities	Yes
				No
				I don’t know
			Down syndrome	Yes
				No
				I don’t know
	17. Single choice	Do you believe that the following factors, if any, increase the risk of birth defects?	Consanguinity*	Yes
				No
				I don’t know
			Usage of certain medications by the mother	Yes
				No
				I don’t know
			Tobacco and alcohol during pregnancy	Yes
				No
				I don’t know
			Infectious diseases	Yes
				No
				I don’t know
	18. Single choice	Do you think that the risk of passing down inherited diseases is less in marriages between distant relatives when compared to marriages between close relatives?^*^		Yes
				No
				I don’t know
Attitudes	19. Likert scale	Please select the degree to which you agree with the following statements:	I would agree/have agreed to marry a relative	Strongly agree
				Agree
				Neutral
				Disagree
				Strongly disagree
			I would encourage my son/daughter to marry a relative of theirs	Strongly agree
				Agree
				Neutral
				Disagree
				Strongly disagree
			I would allow my son/daughter to marry a relative with a known history of genetic diseases	Strongly agree
				Agree
				Neutral
				Disagree
				Strongly disagree
			My parents approve of consanguineous marriages	Strongly agree
				Agree
				Neutral
				Disagree
				Strongly disagree
			I would recommend consanguineous marriages to a friend/relative	Strongly agree
				Agree
				Neutral
				Disagree
				Strongly disagree
			I am willing to undergo premarital testing before getting married	Strongly agree
				Agree
				Neutral
				Disagree
				Strongly disagree
			It is acceptable to have more children after bearing a child with a severe genetic disease	Strongly agree
				Agree
				Neutral
				Disagree
				Strongly disagree
Perceptions	20. Likert scale	Please select the degree to which you agree with the following statements:	I support consanguineous marriages	Strongly agree
				Agree
				Neutral
				Disagree
				Strongly disagree
			I believe that premarital testing is necessary	Strongly agree
				Agree
				Neutral
				Disagree
				Strongly disagree
			I believe legal restrictions should be imposed on couples who have a risk of passing down genetic diseases	Strongly agree
				Agree
				Neutral
				Disagree
				Strongly disagree
			My view of someone would change if I found out they were in a consanguineous relationship	Strongly agree
				Agree
				Neutral
				Disagree
				Strongly disagree
			Children of consanguineous marriages are more likely to get involved in such marriages	Strongly agree
				Agree
				Neutral
				Disagree
				Strongly disagree
	21. Multiple choice	In your opinion, which of the following, if any, are benefits of consanguineous marriages: (select all that apply)		The bride/groom is familiar with the family circumstances
				The financial status of both families is more stable
				It promotes tradition and continuity within the family
				The marriage is more harmonious and loving
				It is easier to raise the children
				If there are any disorders, they are known and expected because the family knows each other
				Consanguineous marriages have no benefits

**Table 7:** Arabic version of the questionnaire Complete Arabic version of the survey instrument used in the study, including items related to inclusion criteria, demographic characteristics, knowledge, attitudes, and perceptions regarding consanguinity. Questions marked with a * are essential knowledge questions, as described in the Methods section

الخيارات	السؤال / التعليمات		رقم البند ونوع السؤال	اسم القسم
نعم	هل تقيم حالياً في دولة الإمارات؟		1. اختيار واحد	الإدماج والاستبعاد
لا				
نعم	هل تجاوز عمرك 18 سنة؟		2. اختيار واحد	
لا				
ذكر	يرجى تحديد جنسك		3. اختيار واحد	المعلومات الديموغرافية
أنثى				
18–29	يرجى تحديد الفئة العمرية		4. اختيار واحد	
30–39				
40–49				
50–59				
60+				
مواطن	يرجى تحديد ما ينطبق عليك		5. اختيار واحد	
عربي مقيم				
غير عربي مقيم				
الشارقة	حدد إمارة إقامتك		6. اختيار واحد	
دبي				
أبو ظبي				
عجمان				
أم القيوين				
رأس الخيمة				
الفجيرة				
لا تعليم	يرجى تحديد أعلى مستوى تعليمي حصلت عليه		7. اختيار واحد	
المرحلة الابتدائية				
المرحلة المتوسطة				
المرحلة الثانوية				
مستوى الدبلوم				
المستوى الجامعي (بكالوريوس)				
الدراسات العليا (ماجستير أو أعلى)				
طالب	يرجى تحديد قطاع / وضعك المهني		8. اختيار واحد	
رعاية صحية				
العمل الاجتماعي / الرعاية				
مهندس / ميكانيكي				
التدبير المنزلي / إدارة القمامة والنفايات				
بائع				
بناء				
خدمات توصيل				
التعليم والبحث				
عاطل عن العمل				
ربة منزل				
متقاعد				
في مجال آخر				
أعزب	يرجى تحديد حالتك الاجتماعية		9. اختيار واحد	
متزوج				
مطلق				
أرمل				
أقارب من الدرجة الأولى	حدد صلة قرابتك بالزوج الحالي / السابق		10. اختيار واحد	
أبناء عمومة من الدرجة الثانية				
قرابة بعيدة				
لسنا أقارب				
لا ينطبق (أعزب)				
أقارب من الدرجة الأولى	حدد صلة قرابة والديك		11. اختيار واحد	
أبناء عمومة من الدرجة الثانية				
قرابة بعيدة				
ليسوا أقارب				
نعم	هل سمعت عن مصطلح زواج الأقارب؟		12. اختيار واحد	لمعرفة
لا				
نعم	هل سمعت عن فحوصات ما قبل الزواج؟		13. اختيار واحد	
لا				
نعم	*هل تعتقد أن أطفال الأزواج الأقارب أكثر عرضة لتشوهات خلقية؟		14. اختيار واحد	
لا				
لا أعرف				
نعم	هل تعتقد أن زواج الأقارب يرتبط باحتمال أكبر لإسقاط الجنين؟		15. اختيار واحد	
لا				
لا أعرف				
نعم	*الثلاسيميا	هل تعتقد أن خطر الإصابة بالأمراض التالية يزداد مع قرابة الزوجين؟	16. اختيار واحد	
لا				
لا أعرف				
نعم	الإعاقة الذهنية			
لا				
لا أعرف				
نعم	الإعاقات السمعية والبصرية			
لا				
لا أعرف				
نعم	متلازمة داون			
لا				
لا أعرف				
نعم	*قرابة الزوجین	ھل تعتقد أن العوامل التالیة، إن وجدت، تزید من خطر الإصابة بعیوب خلقیة؟	17. اختيار واحد	
لا				
لا أعرف				
نعم	تناول الأم لأدویة معینة أثناء الحمل			
لا				
لا أعرف				
نعم	التدخين وشرب الكحول أثناء الحمل			
لا				
لا أعرف				
نعم	الأمراض المعدیة			
لا				
لا أعرف				
نعم	*هل تعتقد أن خطر نقل الامراض الوراثية قد يقل عند الزواج من شخص تجمعك به صلة قرابة بعيدة بالمقارنة بقرابة من الدرجة الأولى؟		18. اختيار واحد	
لا				
لا أعرف				
أوافق بشدة	أوافق او قد وافقت على الزواج من أحد أقاربي	الرجاء تحدید درجة موافقتك على العبارات التالیة	19. مقياس ليكرت	المواقف
أوافق				
حيادي				
أرفض				
أرفض بشدة				
أوافق بشدة	أشجع ابني او ابنتي على الزواج من أحد الأقارب			
أوافق				
حيادي				
أرفض				
أرفض بشدة				
أوافق بشدة	قد أسمح لابني او ابنتي بالزواج من قریب یعاني من الأمراض الوراثیة			
أوافق				
حيادي				
أرفض				
أرفض بشدة				
أوافق بشدة	یوافق والديّ على زواج الأقارب			
أوافق				
حيادي				
أرفض				
أرفض بشدة				
أوافق بشدة	أنصح أصدقائي او أقاربي بزواج الأقارب			
أوافق				
حيادي				
أرفض				
أرفض بشدة				
أوافق بشدة	أنا على استعداد لإجراء فحوصات ما قبل الزواج قبل ان اتزوج			
أوافق				
حيادي				
أرفض				
أرفض بشدة				
أوافق بشدة	من المقبول لزوجین، بعد أنجابھما لطفل یعاني من مرض وراثي خطیر، بإنجاب المزید من الأطفال			
أوافق				
حيادي				
أرفض				
أرفض بشدة				
أوافق بشدة	أنا أؤيد زواج الأقارب	الرجاء تحدید درجة موافقتك على العبارات التالیة	20. مقياس ليكرت	التصور
أوافق				
حيادي				
أرفض				
أرفض بشدة				
أوافق بشدة	أعتقد أن فحوصات ما قبل الزواج ضرورية			
أوافق				
حيادي				
أرفض				
أرفض بشدة				
أوافق بشدة	أعتقد أنه يجب فرض قيود قانونية على الأزواج في حال احتمال نقل مرض وراثي لأطفالهم			
أوافق				
حيادي				
أرفض				
أرفض بشدة				
أوافق بشدة	قد تتغير وجهة نظري لشخص ما إذا اكتشفت أنه متزوج من أحد أقاربه/أقاربها			
أوافق				
حيادي				
أرفض				
أرفض بشدة				
أوافق بشدة	الأولاد من زواج الأقارب هم أكثر عرضة للإقدام على مثل هذه الزيجات			
أوافق				
حيادي				
أرفض				
أرفض بشدة				
الزوجة أو الزوج على درایة بظروف الأسرة	برأيك، أيّ مما يلي، إن وجد، هو من فوائد زواج الأقارب (اختر كل ما ينطبق)		21. اختيار متعدد	
الوضع المالي أكثر استقراراً لكلتا العائلتین				
یعزز التقالید والاستمراریة بین الأسرة				
زواج أكثر انسجاما ومحبة				
سھولة في تربیة الأطفال				
إذا كان ھناك أي أمراض وراثیة، فھي معروفة لدى الأسرة				
لا توجد أية فوائد لزواج الأقارب				
